# Efficient Isolation Method for Highly Charged Phosphorylated Cellulose Nanocrystals

**DOI:** 10.1021/acs.biomac.2c01363

**Published:** 2023-02-07

**Authors:** Marcel Kröger, Olamide Badara, Timo Pääkkönen, Inge Schlapp-Hackl, Sami Hietala, Eero Kontturi

**Affiliations:** † Department of Bioproducts and Biosystems, Aalto University, FI-00076 Aalto, Finland; ‡ Department of Chemistry, University of Helsinki, PB 55, FI-00014 Helsinki, Finland

## Abstract

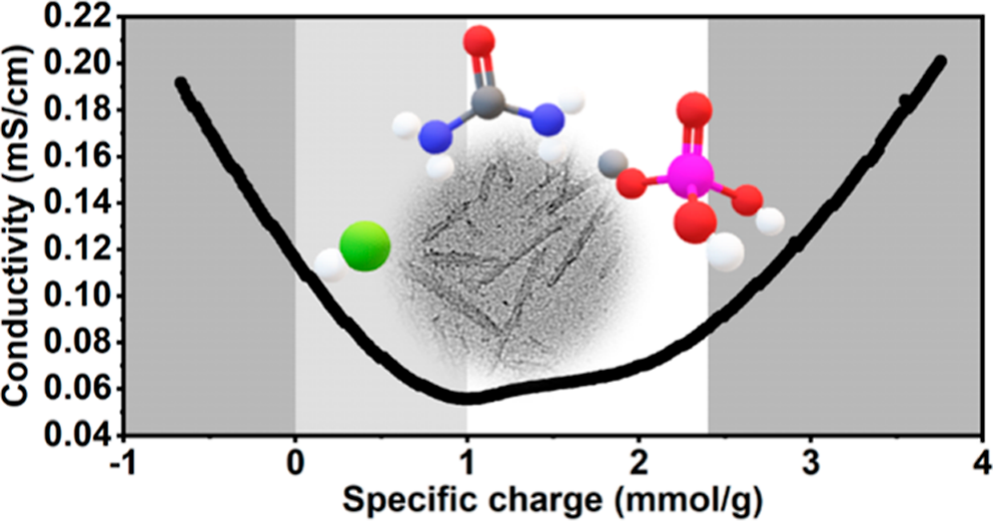

Phosphorylation of cellulose nanocrystals (CNCs) has remained a marginal activity despite the undisputed application potential in flame-retardant materials, sustainable high-capacity ion-exchange materials, or substrates for biomineralization among others. This is largely due to strenuous extraction methods prone to a combination of poor reproducibility, low degrees of substitution, disappointing yields, and impractical reaction sequences. Here, we demonstrate an improved methodology relying on the modification routines for phosphorylated cellulose nanofibers and hydrolysis by gaseous HCl to isolate CNCs. This allows us to overcome the aforementioned shortcomings and to reliably and reproducibly extract phosphorylated CNCs with exceptionally high surface charge (∼2000 mmol/kg) in a straightforward routine that minimizes water consumption and maximizes yields. The CNCs were characterized by NMR, ζpotential, conductometric titration, thermogravimetry, elemental analysis, wide-angle X-ray scattering, transmission electron microscopy, and atomic force microscopy.

## Introduction

Rodlike cellulose nanocrystals (CNCs) have emerged during the past decades as intriguing bio-based nanoparticles with exceptionally widespread applications, including insulating materials, functional coatings, rheology modifiers, membranes for water treatment, and biomedical templates.^1−6^ CNCs can be obtained from purified cellulosic fibers by acid hydrolysis which selectively cleaves the disordered domains in cellulose microfibrils while leaving the crystallites—that is, CNCs—intact.^7−10^ Their colloidal stability in water, however, virtually always requires the presence of charged moieties, such as sulfate, carboxylate, quaternary ammonium, or phosphate groups.^11−13^ As a result, the state-of-the-art of CNC preparation—used by an overwhelming majority both in the industry and in academia—involves the use of concentrated sulfuric acid which simultaneously causes the hydrolysis of the disordered domains and the introduction of sulfate half-esters on the CNC surface.^14^

As highlighted by several recent reviews, the nature of the functional groups on the CNC surface is vital to optimize and fine-tune the material properties on demand.^5,8,10,15^ In this context, phosphorylated CNCs (pCNCs) are attractive because of the specific material properties of phosphorylated cellulose, such as biomineralization, ion exchange, and flame-retardant capabilities.^16,17^ Although phosphate half-esters can in principle be introduced on the CNC surface during hydrolysis with phosphoric acid, it is significantly harder to exert control over the reaction products than it is with sulfuric acid.^16^ Consequently, the attempts to prepare pCNCs by phosphoric acid hydrolysis have ended up with severely lower yields and inferior surface charges with regard to sulfated CNCs.^18^ Mixing phosphoric acid with another mineral acid resulted in only minor improvements.^19^

The classical examples for the phosphorylation of cellulose (not CNCs per se) in the 1940s and 1950s relied not on phosphoric acid but on the use of molten urea in the presence of phosphates.^20−22^ Urea was the key auxiliary which facilitates the reaction as a reaction medium, buffer, catalyst, and/or swelling agent.^20^ However, the detailed reaction conditions were not identified.^22−24^ More recently, the emergence of nanocellulose has led to a revival of the phosphate/urea concept in the production of phosphorylated cellulose nanofibers (pCNFs) where flame-retardancy and ion-exchange properties have been harnessed for the use of modern nanomaterials.^5,24−29^ In addition, pCNFs have exhibited promising potential in promoting biomineralization, biomimicking the formation of the collagen/hydroxyapatite matrix in bones.^16,30,31^ CNCs are a different material from cellulose nanofibers (CNFs), and they are generally applied in a different fashion and/or for different applications than CNFs.^1,2^ In this vein, the production of high charge pCNCs would answer to a demand in modern materials science and technology.

**Figure 1:**
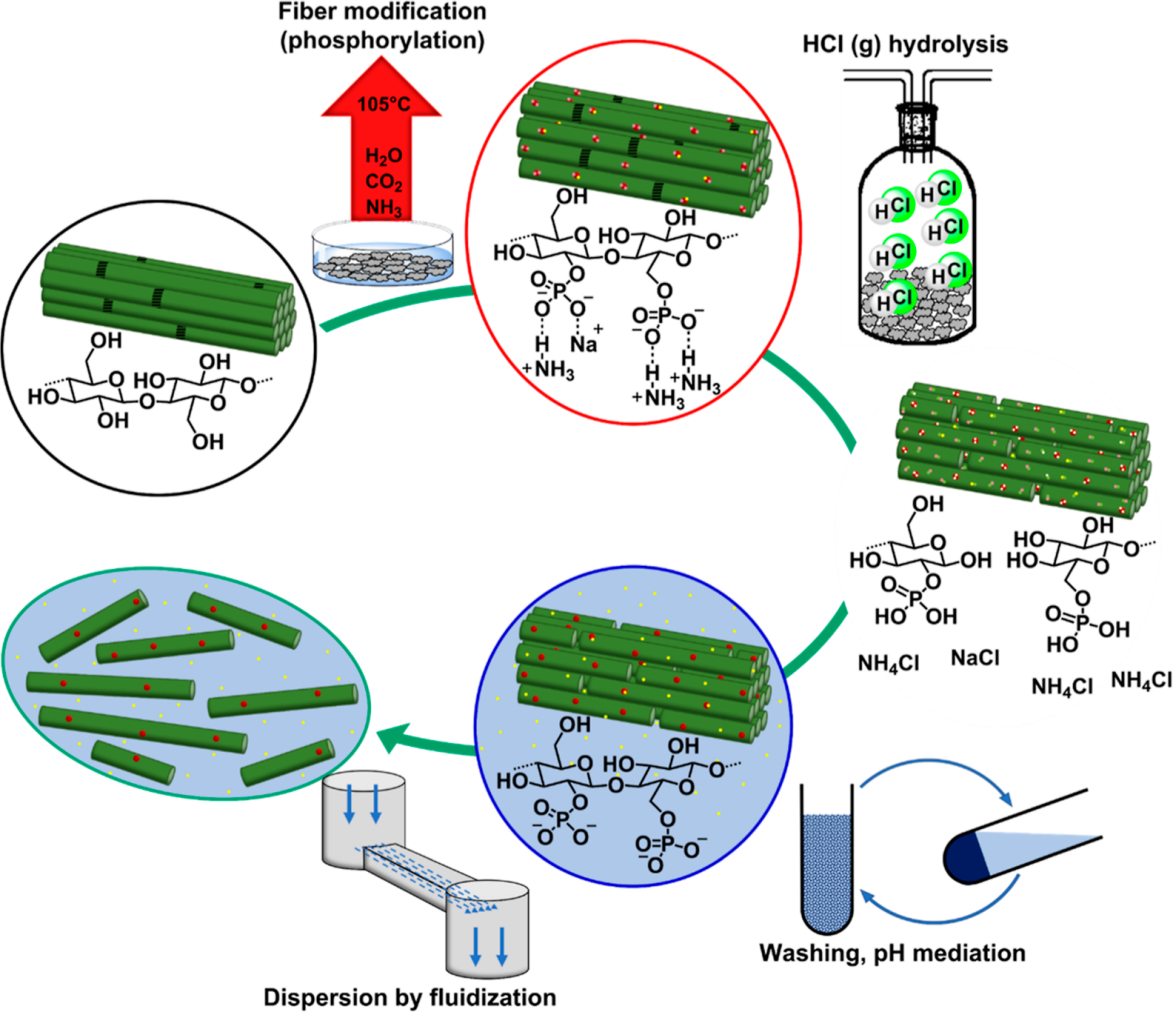
Process scheme: Whatman 1 filter paper is modified with urea/phosphate to yield phosphorylated cellulose fibers. These are subsequently hydrolyzed using gaseous HCl, washed, and dispersed by fluidization.

The past attempts to prepare pCNCs suggest that the presence of water may represent the seminal problem behind the low degrees of phosphorylation. Hydrolysis of cellulose requires water by definition, and the esterification of the phosphate on cellulose hydroxyl groups is severely impeded by water. To this end, we propose a new concept to produce pCNCs where the hydrolysis step has been separated from the phosphorylation step (Figure 1). First, the cellulosic fibers were phosphorylated in a urea/phosphate mixture. Second, the modified fibers were hydrolyzed by gaseous HCl which—unlike the aqueous hydrolysis routes—requires minimal purification after the reaction. The water for the reaction is provided by the small amounts of moisture absorbed on the fibers under ambient conditions.^32−34^ The protocol entirely omits the tedious process control required for phosphoric acid in solution. Furthermore, it significantly reduces both the water and energy consumption compared to the traditional approach of modifying CNCs after their aqueous hydrolysis as no workup or traditional drying step is required for modification and hydrolysis. Importantly, an up to 20-fold increase in the (mono)phosphate content of the pCNCs was achieved in contrast to the published studies. Altogether, the method enables the production of a completely new type of pCNCs that pave way for entirely novel material applications in the future.

## Materials and Methods

### Materials

Whatman 1 filter paper (catalog number WHA1001125, 125 mm diameter), phosphoric acid (H_3_PO_4_, 85% aqueous solution, CAS 7664-38-2, VWR chemicals), sodium monophosphate dihydrate (NaH_2_PO_4_·2 H_2_O, CAS 13472-35-0, 98%, Supelco), urea [CO(NH_2_)_2_, >99%, CAS 57-13-6, Sigma-Aldrich], sodium chloride (NaCl, >99%, CAS 7740-23-5, VWR chemicals), sodium hydroxide (NaOH, >99%, CAS 1310-73-2, VWR chemicals), HCl gas (99.8%, CAS 7647-01-0, AGA), sulfanilamide (OAS, CAS 63-74-1, Elemental Microanalysis Ltd), nitric acid (HNO_3_, 67–69% Assay, CAS 7697-37-2, Romil Chemicals Ltd), hydrochloric acid (HCl, 34–37% Assay, CAS 7647-01-0, Romil Chemicals Ltd), hydrofluoric acid (HF, 40%, guaranteed reactant, CAS 7664-39-3, Merck), and Milli-Q water (18.2 MΩ cm resistivity) were used without further purification. Sodium hydroxide solution (NaOH, 1 M, CAS 1310-73-2, Titripur Reag., Merck) was diluted to a concentration of 0.1 M with degassed Milli-Q water and used for conductometric titrations. sCNCs were prepared from Whatman 1 filter paper by sulfuric acid hydrolysis (8.75 mL 64% H_2_SO_4_ per 1 g Whatman 1 filter paper, 45 min, 45 °C), as described elsewhere,^14^ and by means of elemental analysis found to contain 0.194 mmol/g sulfate.

### Cotton Fiber Modification

In a typical experiment, 3.15 g of dry Whatman 1 cotton linter filter paper (three sheets) were wetted in 500 mL of water and blended^35^ with a Braun 300 W hand blender until no chunks could be observed anymore. Vacuum filtration was then used to concentrate the obtained pulp, yielding 15 g of wet fibers.

For the modification of the fibers, we adapted the procedure used by Rol et al. to produce pCNF.^24^ As such, in a typical experiment, 1.10 g of phosphoric acid (85%), 1.49 g of monosodium phosphate dihydrate, and 5.5 g of urea were added to the wet fibers (AGU:H_3_PO_4_:NaH_2_PO_4_:urea 1:0.5:0.5:4.7), along with deionized water to bring the total weight to 40 g. After careful mixing, the samples were oven-dried in aluminum crucibles for 72 h at 105 °C. A slight brown discoloration was observed on the pulp surface while the bulk remained colorless (Figure S1).

### HCl Gas Hydrolysis of Modified Fibers

The HCl gas hydrolysis was conducted in the custom-built reactor assembled and first described by Pääkkönen et al.^34^ The modified pulp was blended again in the dry state to increase the surface area. Then, it was transferred into a 1 L reactor, to which 1.1 bar HCl was added at room temperature. The total weight gain of the reactor system due to HCl addition, which both physically adsorbs on the pulp and reacts with deprotonated phosphate groups, was 2.6 g. The mixture was left to react for 4 h, before the overpressure was released, and the reactor flushed with air for 15 min to expel the lingering and slowly desorbing HCl. During the hydrolysis, the discoloration of the pulp intensified slightly, but no other changes were observed.

### Washing

The dry, modified, and hydrolyzed pulp was wetted again in 80 mL of deionized water, resulting in pH 1.2, and 1 M NaOH solution was added until pH 6.5 was reached. The aim is to wet the fibers completely at a neutral pH to prevent the hydrolysis of the imparted surface esters and stop the cellulose hydrolysis by the lingering residual HCl. The resulting mixture was stirred overnight to promote wetting and homogenization. Then, the pulp was centrifuged at 9000*g* relative centrifugal force for 10 min, decanted, and resuspended in 300 mL deionized water. In order to remove soluble hydrolysis byproducts, the 300 mL of suspension was stirred for 30 min, before being subjected to the same centrifugation and resuspension procedure. The pH and electrical conductivity of the decanted aqueous phases were monitored after each step. A total of four washing steps were required to reach conductivities below 50 μS/cm and a pH of 8.9.

Alternatively, after wetting the modified and hydrolyzed pulp by stirring overnight at pH 6.5 and centrifuging and decanting, the pulp was acidified by suspending it in 300 mL of 1 M HCl to protonate the phosphate groups, displacing the unwanted counterions. Further washing was conducted following the stirring, centrifuging, decanting, and redispersion in water routine until conductivities below 50 μS/cm and a pH of 4.5 were reached.

### Conductometric Titration of Pulp

Conductometric titrations were carried out on the washed pulp, according to the protocol described by Ghanadpour et al.^25^ A sample of the wet, washed pulp, containing 300 mg of cellulose if dried, was added to 500 mL of degassed Milli-Q water and 0.5 mL of 0.5 M NaCl solution. The mixture was acidified with 5 mL of 0.1 M HCl solution and titrated with 20 mL of 0.1 M NaOH solution at 0.1 mL/min.

### Dispersion of Nanocrystals

The produced CNCs were dispersed using a Microfluidics M-110P microfluidizer. The washed pulp was suspended in water at a concentration of 1 wt % and passed three times at 1500 bar through a pair of Z-type collision chambers with dimensions of 400 and 200 μm, respectively. The obtained dispersion was filtered through a Sefar Nitex 03–10/2 woven open mesh fabric (PA 6,6, 10 μm openings, 2% open area) to exclude large aggregates if present. A CNC yield of 70% was achieved based on the mass of cellulose in the modification step and the obtained CNCs (92.2 wt % cellulose, 7.8 wt % phosphate groups).

### Zeta Potential

ζpotentials were measured using a Malvern Zetasizer ZS90. According to the protocol proposed by Foster et al.,^36^ pCNC dispersions were diluted to 0.1 wt %. The pH was adjusted by adding 0.1 M HCl or NaOH solutions. Given the ambivalence in the literature as to whether to adjust the ionic strength of the analytes^18,36^ or not,^19,23,37^ to be able to compare the results, the ζpotential of the pH-adjusted analytes was measured both before and after adjusting the ionic strength to 5 mM by NaCl. This adjustment affects the pH through the screening of the electrostatic double layers of the particles and thereby the dissociation of the surface groups, causing the pH values to vary slightly between both measurements.

For each sample and ionic strength, three measurements were performed to obtain the electrophoretic mobility of the analyte particles. The ζpotential was calculated using Smoluchowski theory, which is valid only for spherical particles. The obtained values, therefore, are representative, not absolute.

### AFM

Silicon substrates were immersed in a 3.5 wt % PEI solution (*M*_w_ = 2000–4000 g/mol) for 15 min, rinsed carefully with deionized water, and air-dried. Subsequently, 50 μL of 0.01 wt % pCNC dispersion was spin-coated at 4000 rpm. The substrates were imaged using a Bruker Multimode 8 AFM in the tapping mode. Cantilevers of the model NCHV-A by Bruker with force constants of 42 N/m and 320 kHz resonance frequency were used. The obtained images were baseline corrected by plane-fitting and flattened using NanoScope Analysis 1.5 software. The height of the individual particles was analyzed using the particle analysis function of the same software on individually selected particles, whereas the length of individual particles was measured manually, using ImageJ. Overlapping particles were considered for neither height nor length analyses.

### Transmission Electron Microscopy (TEM)

Samples were prepared dropping 5 μL of 0.01 wt % pCNC dispersion onto formvar/carbon-supported copper grids (size 300 mesh, Sigma-Aldrich) that had been decontaminated for 10 min, using a Fischione model 1070 NanoClean device, to remove contaminants and thereby increase hydrophilicity. The samples were left to dry in air for 5 min and then blot-dried. The substrates were imaged using a JEOL JEM 2800 HR analytical TEM in the bright-field mode.

### Wide-Angle X-ray Scattering (WAXS)

Wide-angle X-ray scattering (WAXS) data were obtained using a Xenocs Xeuss 3.0 SAXS/WAXS system (Xenocs SAS, Grenoble, France). The system consists of a microfocus X-ray source (sealed tube) with a Cu target and a multilayer mirror which yields a parallel beam with a nominal wavelength of 1.542 Å (combined Cu K-α_1_ and Cu K-α_2_ characteristic radiation). The source operates at 50 kV and 0.6 mA. The beam is collimated by a set of variable slits, and the experiments were conducted with a beam size of 0.7 mm. As the system does not include a beam stop, direct measurements of sample transmission were conducted. The data were acquired using an area detector (Eiger2 R 1M, Dectris AG, Switzerland). The sample-to-detector distance was calibrated by measuring the diffraction from a known LaB_6_ standard sample.

Freeze-dried cellulose samples were analyzed by sealing the analyte in aluminum washers using Kapton films. Scattering contributions from the empty chamber and the two layers of Kapton films were determined by measuring an empty washer under the same conditions and were subsequently subtracted from the azimuthally averaged data.

### Elemental Analysis

The cellulose samples were freeze-dried and kept in a desiccator overnight to exclude as much moisture as possible. Analyses for carbon, hydrogen, and nitrogen contents were carried out on a Thermo Scientific FlashSmart CHNS/O elemental analyzer equipped with a copper reduction phase. 2 mg of cellulose was burnt in a folded tin crucible in an oxygen atmosphere, whereupon helium was used as a carrier gas. The obtained chromatograms were analyzed using EagerSmart software by Thermo Scientific. Sulfanilamide was used as a calibration standard.

Phosphorus was determined by digesting the pCNCs in accordance with the standard ISO 14869-3:2017. A microwave-assisted digestion via an acid mixture of nitric acid (HNO_3_), hydrofluoric acid (HF), and hydrochloric acid (HCl) was conducted. The obtained solutions were analyzed by means of inductively coupled plasma optical emission spectrometry (ICP-OES) using an Agilent 5900 SVDV system. The phosphorus content was quantified at a wavelength of 213.617 nm.

### NMR Analysis

^31^P solid-state magic angle spinning (MAS) NMR was measured on a Bruker Avance III spectrometer operating at a ^1^H frequency of 500 MHz (observed resonance frequency for ^31^P of 202 MHz) using 4 mm ZrO_2_ rotors spun at 13 kHz. Proton decoupling was performed by means of SPINAL-64 decoupling. In order to compare the results to the previously published data, the conditions outlined by Fiss et al.^17^ were applied. As such, 128 scans were collected for each sample with a recycle delay of 150 s.

### Thermogravimetry

The thermal decomposition of the produced samples was analyzed using a Netzsch STA 449 F3 Jupiter analyzer. The freeze-dried samples (5 mg each) were heated in 85 μL aluminum oxide crucibles (Netzsch) from 40 to 900 °C with a heating rate of 10 K min^–1^ in a stream of 50 mL/min air and 20 mL/min nitrogen (70 mL/min gas flow consisting of 15 vol % oxygen).

## Results and Discussion

### Modification and Hydrolysis of Cotton Fiber

**Figure 2:**
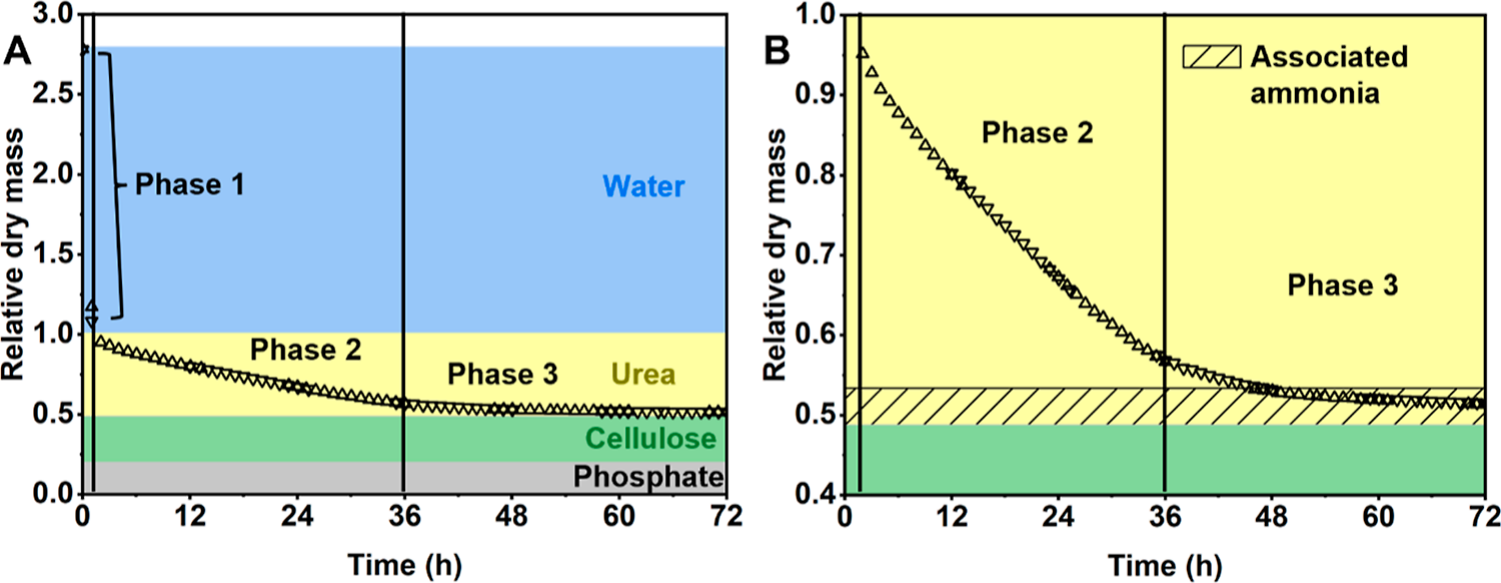
Decrease in relative reacting mass over the course of the modification reaction. The reactant mass was normalized to the initial dry matter content. Upward and downward pointing triangles indicate two separate data sets. The background represents the separate reactants (A). Noticeable changes in the rate of weight loss appear after 36–55 h, indicating the completion of the urea decomposition and modification reactions (B).

**Scheme 1:**
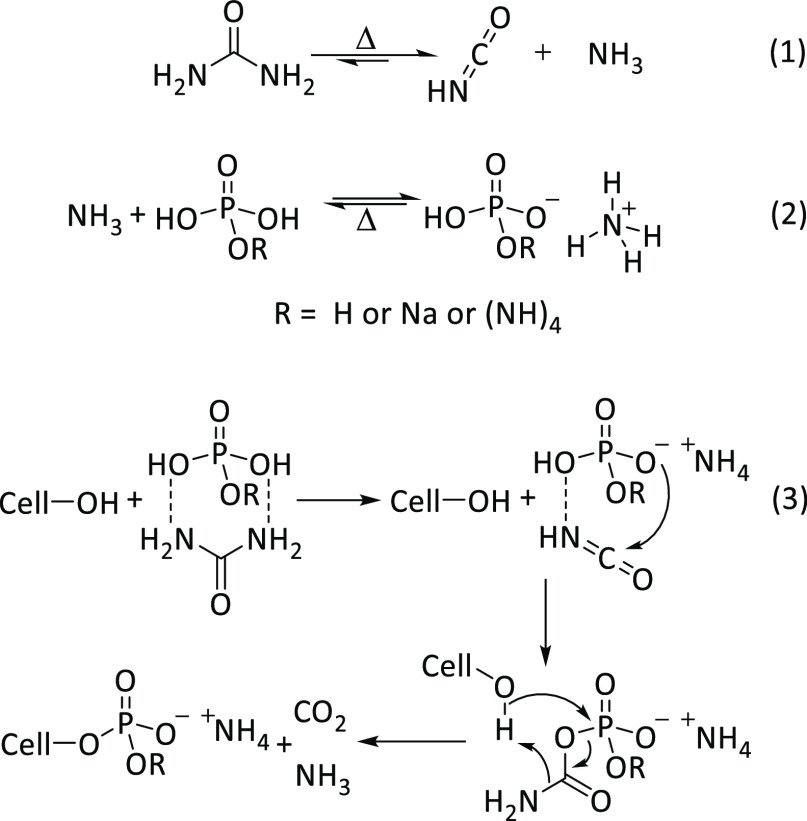
Decomposition of Urea into Isocyanic Acid and Ammonia (1), Thermally Reversible, Neutralization of the Phosphoric Acid by the Released Ammonia (2), and a Plausible Pathway to Cellulose Phosphate (3)

At elevated temperatures and in dry conditions, urea decomposes primarily into isocyanic acid and ammonia^38^ which then associates to the partially neutralized phosphoric acid, forming sodium ammonium phosphates. Crucially, the decomposition products form the reactive intermediates that eventually yield the cellulose phosphates (Scheme 1). We emphasize, however, that the genuine reaction pathway has remained unidentified till date. To monitor the crude kinetics of a complex system, the reaction mixture was simply weighed at regular intervals for a 72 h period (Figure 2), similar to a study by Noguchi et al. who used the same approach in following pCNF formation.^39^ Isocyanic acid is volatile at the reaction temperature (105 °C) and although it can participate in a number of further reactions,^38,40^ it appears to diffuse out of the system as the weight loss in the reaction mixture after water removal (Phase 1) is linear with time (Phase 2, see also Figure S2 for a linear fit). Consequently, the reaction order appears to be zero until the urea is fully decomposed (end of Phase 2 after 36 h). At this point, the phosphoric acid is fully neutralized to dibasic (NH_4_)_2_HPO_4_ by the released ammonia, and labile reaction products of isocyanic acid remain to decompose in Phase 3. Eventually, the only labile compound left is (NH_4_)Na_x_H_(2–*x*)_PO_4_. Analogous to acid hydrates, ammonium salts can undergo thermolysis and loose volatile ammonia. At 100 °C, the vapor pressure of ammonia from diammonium phosphate is 12.1 hPa and thus not negligible.^41^ Consequently, once the urea has fully decomposed and ammonia is released from the phosphate, the mixture becomes more acidic. This acidification in turn promotes the degradation of the cellulose substrate, which results in the increasing discoloration of the samples after long reaction times, as shown in Figure S2.

The actual mechanism of the phosphorylation reaction that is routinely being cited in the literature^23,24,42^ refers to a publication by Nehls and Loth.^43^ It postulates a hexagonal transition state involving cellulose, phosphate, and urea and suggests that the reaction proceeds by three concerted transitions: the protonation of urea by phosphoric acid, a nucleophilic attack by phosphate on the cellulose C6-atom, and the elimination of hydroxide in an S_N_2-type transition. The released hydroxide would then neutralize the protonated urea to yield cellulose phosphate, water, and urea. This concept, although widely cited, has some rather obvious shortcomings. Phosphoric acid is not strongly acidic enough to protonate urea, phosphate is not a nucleophile, and the formation of phosphate esters is generally accepted to occur via nucleophilic substitution on the phosphorus, not the carbon atom.^44^ Furthermore, the suggested mechanism fails to explain why urea is necessary in the process, why ammonium cannot catalyze the same transition, or why the decomposition of urea is necessary for the phosphorylation reaction.

Given these limitations, we hypothesize instead that the modification reaction occurs by activation of the phosphate as an intermediary carbamoyl phosphate, as shown in reaction (3) of Scheme 1. Carbamoyl phosphate is formed by the addition of isocyanic acid to phosphoric acid.^45^ This activated phosphate will then undergo nucleophilic substitution by cellulose hydroxyl groups, resulting in the elimination of a carbamate anion.^44^ This anion can decompose into carbon dioxide and ammonia, suppressing a potential reverse reaction under the given reaction conditions. Furthermore, carbamoyl phosphate has been shown to undergo thermal decomposition well below 150 °C, yielding both phosphate and pyrophosphate.^46^ In contrast to the commonly cited transition state, this activation sequence would in fact explain the requirement for decomposing urea as an auxiliar, the stalling of the reaction in the presence of ammonium phosphate instead of urea phosphate, and the formation of phosphate esters and diphosphates at reaction temperatures below 150 °C, while fully adhering to the concept of Lewis acids and bases. We have not carried out any monitoring other than gravimetry over the course of the reaction to try and confirm this mechanism, however. This could be subject to further studies.

While the modification reaction is usually carried out at 150 °C, lowering the temperature to 105 °C allows for a better process control at the expense of longer reaction times (see, Table S1). This is due to the significantly slower degradation of urea below its melting point, which reduces the concentration of reactive intermediates needed for the phosphorylation reaction. However, the lower temperature also significantly reduces the degradation of cellulose by dehydration or oxidation (see, the visual consequence in Figure S2). Seminally, ammonium phosphate has been shown to degrade above 150 °C, eliminating ammonia and releasing the free acid, which, at elevated temperatures, can further undergo condensation reactions to form pyrophosphates.^41,47^ In high concentrations, orthophosphoric acid and more strongly acidic pyrophosphates promote the degradation of cellulose, so it is of interest to slow down their formation by decreasing the reaction temperature.

Apart from shedding light on the reaction kinetics, Figure 2 emphasizes the reproducibility of the modification reaction, illustrating a deviation of the mass of the two investigated samples of less than 1% at all coinciding datapoints. Furthermore, a change in the reaction rate can be found after 36 h (onset of Phase 3). Further studies are needed to clarify what causes this termination of the steady state. Possible causes are the conclusion of urea degradation or a change in reactant concentrations.

Our method relies on the complete degradation of the added urea which accounts for the long reaction times. If the reaction time is cut short, side reactions occur upon the eventual contact with HCl gas in the second step of the process. The effect is an almost instantaneous, irreversible blackening of the cellulosic material, as displayed in Figure S3. The fact that no such blackening can be observed after long reaction times during the modification step indicates that this phenomenon is tied to either urea or its degradation products. However, this side reaction can be avoided completely by ensuring the completion of the modification reaction.

The hydrolysis of the cellulose with gaseous HCl causes a significant drop in the degree of polymerization (DP), as indicated by the obtained data from viscometry shown in Table S2. The method relies on the adsorption and dissociation of HCl molecules into the nanolayer of moisture that is present even in dried cellulose.^33^ This results in an exceptionally low surface pH of the cellulose fibers, which facilitates and catalyzes the hydrolysis reaction predominantly in the disordered regions. The theoretical minimum water content for the hydrolysis reaction to occur is one water molecule per one chain scission. Given the viscosity-average DP, this amounts to roughly 0.03 wt % of water with respect to the cellulose. The water content of cotton linters kept under atmospheric conditions is usually in the order of ca. 5 wt %.^34^ However, in our case, in the presence of phosphate salts, which form stable monohydrates below 100 °C when exposed to moist air, the moisture content of the mixture was 6.5 wt %. This is based on the weight increase between drying at 105 °C and blending in the reaction mixture prior to the hydrolysis. This means that in relation to the cellulose in the system, the water content amounts to 11 wt %., well exceeding the theoretical minimum of 0.03 wt %.

After phosphorylation, the hydrolysis reaction required the adsorption of a slightly larger amount of HCl gas compared to the hydrolysis of neat cellulose, which is due to the protonation of partially neutralized phosphate groups (see, Figure 1). It was found that the adsorbed HCl after flushing the reactor with air amounted to 0.17–0.3 g/g cellulose rather than 0.07–0.08 g/g reported for pristine cellulose.^34^ As such, per gram cellulose, 0.09–0.22 g (2.5–6.3 mmol) of HCl is consumed in the protonation of the phosphate. This corresponds well to the amount of phosphate introduced to the system (6.4 mmol/g cellulose, containing 3.2 mmol monobasic phosphate) and indicates that by flushing with air, most of the unreacted and unabsorbed HCl can be expelled from the system and potentially be recycled.

Given the retention of some of the HCl, the dry modified, hydrolyzed fibers were suspended in water and neutralized to pH 6.5 to stop the hydrolysis reaction. The neutralization also significantly facilitates the complete wetting of the fibers, which is, of course, vital to removing byproducts and contaminants. The thus-obtained gel contains modified, hydrolyzed, neutralized cellulose fibers as well as excess phosphates and sodium and ammonium chloride. Based on the weight of the reaction mixture after the modification step and the amount of HCl that remained in the system after hydrolysis, the ratio of sodium/ammonium is expected to be larger than 3:1. Still, the significant amount of ammonium in the system needs to be removed as it would interfere with the following titrimetric analyses.

**Scheme 2:**
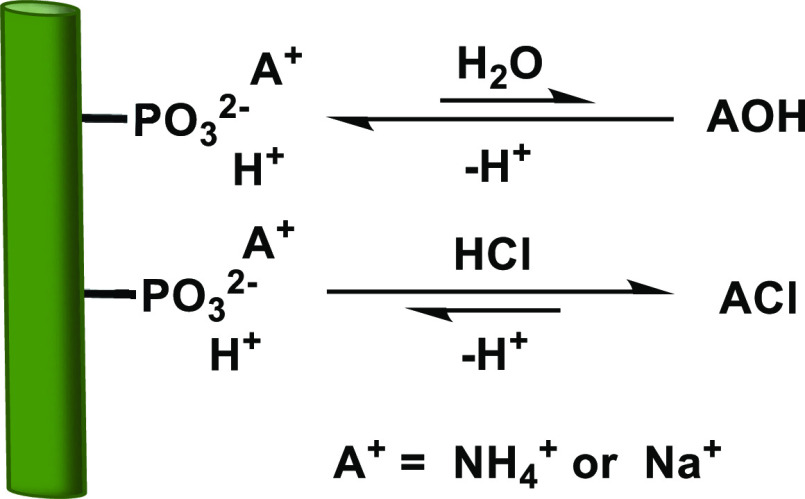
Cation Retention on the Substrate: Washing Following Partial Neutralization Results in the Replacement of Counterions with Protons from Water, which Causes the pH of the Washing Solution to Rise (pH 8.9) At this point, the backward reaction of deprotonating the phosphate groups again becomes favorable. Washing under acidic conditions results in full protonation (final pH 4.5), facilitating quantitative counterion removal

During washing by centrifugation, the hydrolyzed cellulosic substrates behaved like ion-exchange materials, as shown in Scheme 2. As such, excess salt could easily be removed, but counterions were retained on the fibers. This is the reason behind the increase in pH of the washing solution. Following the principles of electroneutrality, the removal of counterions is only possible if the charge is compensated. Therefore, as the counterions migrate from the surface into the (pure) washing water, protons compensate their charge on the particle surface. The result is the net increase in pH. The increased pH will then promote the deprotonation of the surface groups, resulting in a dynamic equilibrium at pH 8.9.

However, when the phosphate groups were protonated at the beginning of the washing cycle, the excess ions could be removed successfully, until the mixture was essentially ion free, which is indicated by minimal conductivities (below 50 μS/cm) of the washing water. The pH increased to 4.5 due to the deprotonation of the strongly acidic first protons of a fraction of the surface phosphates. Again, the dissociation and reprotonation of the surface groups are in dynamic equilibrium.

Either state could be reached within four centrifugation steps, after which no further decrease in conductivity or change in pH could be observed. Thus, the total water consumption per gram of produced CNCs amounted to 250 mL for the fiber homogenization, 20 mL for the modification reaction, 900 mL for washing, and a further 100 mL for the dispersion, totaling at 1.27 L/g CNCs. This could be further reduced to 0.6 L/g CNCs when the synthesis was successfully scaled up to 30 g of cellulose instead of 3 g.

### CNC **Properties**

pCNC properties and their comparison to literature values are presented in Table 1. It clearly shows the significant difference in surface charges achieved by phosphorylation in aqueous solution compared to those obtained by the reaction with urea phosphate in the absence of water. Evidently, this does not depend on the cellulose source as similar degrees of phosphorylation are obtained by the same method across all cellulosic substrates. Instead, it is the combination of urea as an auxiliar and the absence of water that enable the improved phosphorylation yields. This is illustrated by the mixed-acid approaches by Amin et al.^19^ The surface esterification reaction during phosphoric acid hydrolysis is acid-catalyzed. This means that the yield is not dependent on the pH of the solution, which is evident considering the low surface charge of the obtained CNCs. Nevertheless, the lower pH enables higher reaction rates, that is, a higher reactivity of the phosphate. The fact that the degree of phosphorylation still did not increase must therefore be down to the water in the system and its unfavorable impact on the equilibrium of the esterification reaction. This is why the modification of CNFs by phosphate salts shows significantly higher yields (Naderi et al.^50^) which are improved further by the addition of urea (Rol et al.^24^). Still, given the lack of acidic strength in the urea phosphate mixtures, no hydrolysis of the cellulosic fibers to isolate CNC can take place. This problem was overcome here by introducing the gaseous HCl hydrolysis as a consecutive treatment. We obtained the expected degree of phosphorylation despite the HCl treatment, which means that the hydrolysis of the dry fibers, unlike the hydrolysis in aqueous suspension, is orthogonal to the modification reaction.

**Table 1:** Zeta Potential, Surface Charge, and Phosphate Content Reported for pCNCs (Upper Part) and CNFs (Lower Part) Compared to This Work

product	cellulose source	reagent	ζpotential (mV)	surface charge (mmol/kg)	phosphate content (mmol/kg)	ref
pCNC	Whatman 1 filter paper	urea/NaH_2_PO_4_/H_3_PO_4_	–35 to–45	1920^a^	1000^e^	this work
pCNC	Whatman 1 filter paper	H_3_PO_4_(aq)		10.8^a^	3.95^b^	(35)
pCNC	coffee grounds	H_3_PO_4_(aq)		48.4^a^	25.8^d^	(48)
pCNC	tomato plant residue	H_3_PO4 (aq)	–36.9	79.2^a^		(37)
pCNC	Whatman ashless filter aid	H_3_PO_4_(aq)	–9.8 to–17.3		8.2–44.5^b^	(6)
pCNC	MCC Avicel PH-101	H_3_PO_4_:H_2_SO_4_ 4:1(aq)	–33.2	98^a^		(19)
		H_3_PO_4_:HCl 4:1(aq)	–38.9	102^a^		
pCNC	giant Reed plant CMF	H_3_PO_4_(aq)		254^a^		(49)
pCNC	mechanically individualized wood CNC	H_3_PO_4_ (aq)	–25 to–30		383,^b^ 435^f^	(23)
		H_3_PO_4_ in molten urea	–25 to–35		1213^b^1038^f^	
pCNC	commercial wood CNC	H_3_PO_4_ (aq)			1200^c^	(17)
		Urea/H_3_PO_4_ (aq)			1600^c^	
		Urea/P_4_O_10_			3300^c^	
pCNF	uncharged CNF	H_3_PO_4_	–25 to–40		16^b^ 67^f^	(23)
		urea/H_3_PO_4_	–30 to–40		1370^b^1173^f^	
pCNF	dissolving pulp	Urea/(NH_4_)_2_HPO_4_		1840^a^		(25)
pCNF	dissolving pulp	NaH_2_PO_4_		730–2030^a^^,^^g^	310–960^e^^,^^g^	(50)
pCNF	sugarcane bagasse	Urea/(NH_4_)_2_HPO_4_		1920–2560^a^		(51)
pCNF	eucalyptus bleached kraft pulp Fibria T35	Urea/(NH_4_)_2_HPO_4_		2930^a^		(24)
pCNF	softwood pulp	Urea/(NH_4_)H_2_PO_4_			230–2200^b^	(39)

a Determined by conductometric titration.

b Determined by molybdate colorimetric essay.

c Determined by solid-state NMR.

d Determined by XPS.

e Determined by elemental analysis (see, Table S3).

f Determined by potentiometric titration.

g Calculated from reported values for degrees of substitution.

The highest degrees of phosphorylation of cellulose were obtained by Fiss et al.,^17^ who followed up on the urea phosphate approach by employing condensed phosphates and mechanochemistry. The obtained NMR spectra may indicate that in the absence of solvents, the direct grafting of condensed phosphates by the same method is feasible just as well, increasing the phosphate content significantly.

**Figure 3:**
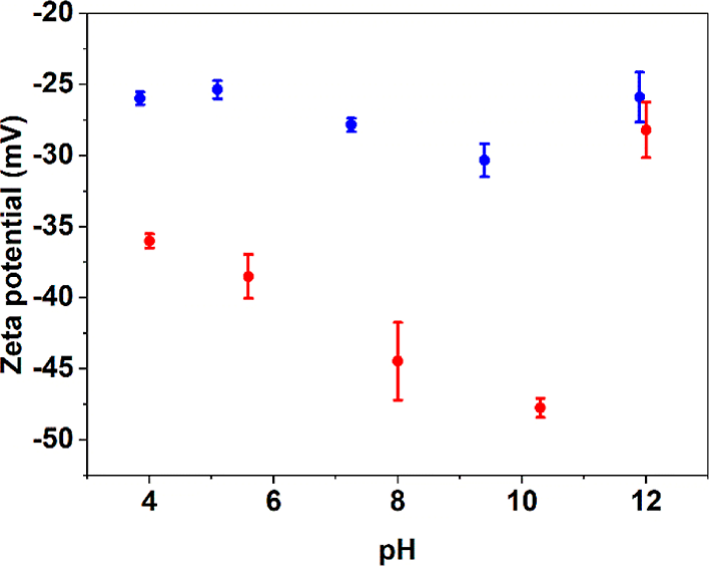
ζpotential of the produced pCNC at varying pH values at low ionic strength without electrolytes (red) and in 5 mM NaCl (blue).

It should be noted, however, that data in Table 1 are not unequivocally comparable. First, with the listed ζpotentials, it is not always clear in the literature references whether the ionic strength has been adjusted or not. The electrophoretic effect, the internal field effect, and the relaxation effects all cause the particle mobility to drop at increased ionic strengths in a nonlinear fashion. As the particle mobility is the quality monitored in the electrophoretic measurements, the deduced ζ-potential values are indisputably affected by the ionic strength. The effect is experimentally demonstrated with our pCNC samples in Figure 3: the absolute values of ζpotential are systematically lower after 5 mM addition of NaCl as a background electrolyte. The adjusted ionic strength is also the likely reason behind the noticeably lower ζpotential determined by Vanderfleet et al. (Table 1).

The second—intrinsically related—issue with ionic strength is its effect on the dissociation behavior of the charged groups during conductometric titration. Besides the obvious decrease in ζpotential, Figure 3 shows that the pH is decreased upon higher ionic strength—presumably due to competition between H^+^ and Na^+^, impeding the interactions between the protons and the phosphate groups. These effects are common for polyelectrolytes which do not dissociate fully once the charge density exceeds a critical value, causing a significant drop in conductivity compared to the corresponding ions in solution (see also Figure S4). Specifically with high charged pCNCs, as the dissociation of the strong acid proton no longer occurs spontaneously but at an apparently higher p*K*_a_ value, the dissociation of the second proton occurs simultaneously with the condensation of Na^+^ on the charged surface. These processes cannot be separated from each other by simple linear regression of the titration curve. While models for the titration curve exist for various polyelectrolyte solutions, there are no corresponding solutions for nanoparticle dispersions.

Nevertheless, we have calculated the surface charge values for pCNCs by linear extrapolation for comparison, as listed in Table 1. We are also currently working on how to extract accurate values from the conductometric titration of CNCs in general, and the results will be published later elsewhere. For now, it should be noted that our pCNCs show the same neutralization behavior as pCNF produced by the same modification protocol, indicating similar surface charges as supported by the results of the elemental analysis.

**Figure 4:**
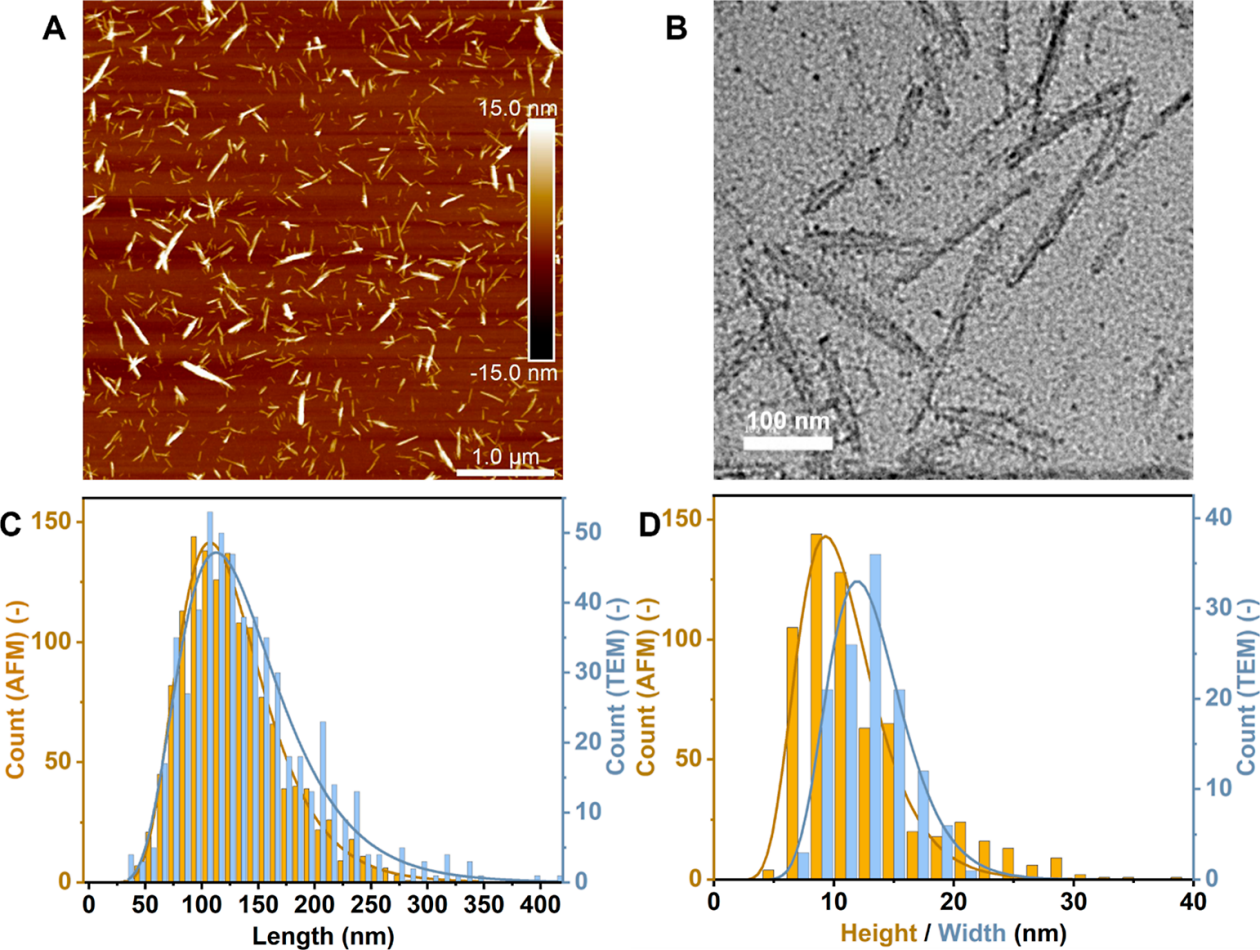
AFM height image of the produced pCNCs (5 × 5 μm^2^) (A), TEM image of the produced pCNCs (scale bar represents 100 nm) (B), length distribution histogram of pCNCs (C), retained from image analysis of the AFM and TEM images and height and width distribution (D). The distributions were obtained from a total of five AFM pictures and eight TEM pictures of the same magnification as in (A) and (B), respectively.

**Figure 5:**
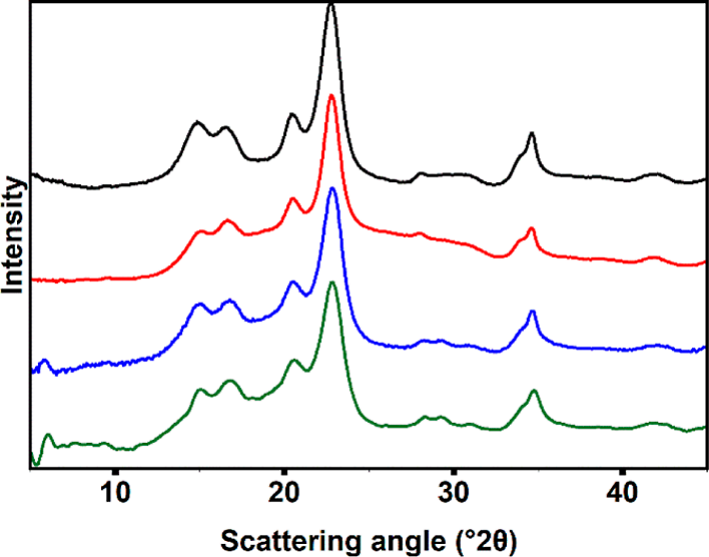
WAXS diffractogram of Whatman 1 cotton linters (black) modified cellulose (red), hydrolyzed, and washed phosphorylated pulp (blue) and dispersed pCNCs (green). The scattering patterns correspond to cellulose *I*_β_, and the cellulose remains crystalline throughout the process.

The sizes and crystallinity of our pCNCs were investigated by means of AFM and TEM as well as WAXS. The results are shown in Figure 4 and 5 and Table 2.

**Table 2:** Dimensions and Aspect Ratio of Our pCNCs

	analysis technique	
aspect	TEM	AFM
length	130.6 ± 52 nm	120.7 ± 43 nm
width	12.7 ± 3.3 nm	
height		10.3 ± 3.4 nm
aspect ratio	10.2 ± 6.7 nm	11.7 ± 8.0 nm

Both AFM and TEM analyses show that nanocrystals have been isolated successfully from the phosphorylated pulp (see, Figure 4). The average length of the nanocrystals is roughly 120 to 130 nm. The length distributions found by AFM and TEM analysis are in good agreement with each other as the difference in the obtained average lengths is significantly smaller than the respective standard deviations. Comparing the length to the average width, as obtained by TEM (12.74 ± 3.3 nm), yields an aspect ratio of 10.26 ± 6.74. The average height of the particles was found to be slightly lower than the average width. This presumably originates from the parallel nature of the crystal aggregates in CNCs: they are lying flat on the substrate, and AFM height measurement is therefore able to probe the height of a single crystal, not the width of the aggregate. TEM, in turn, is able to determine the real width of the aggregate consisting of parallel crystals.^52^

Previously reported pCNCs extracted from Whatman 1 filter paper by phosphoric acid hydrolysis have led to significantly longer particles. As such, Camarero et al. found the dimensions of their particles to be 317 nm by 31 nm (aspect ratio 11)^35^ while Vanderfleet et al. isolated particles with lengths between 238 nm and 475 nm during their optimization study.^18^ Our pCNCs are significantly shorter and thinner than that, which can be attributed to HCl being a stronger acid than phosphoric acid. The length distribution of our pCNCs perfectly matches previously reported dimensions for CNCs isolated by HCl vapor from the same source.^33^ Similar conclusions can be drawn for the mixed-acid hydrolysis approaches. Amin et al. reported 363–425 nm by 17–22 nm (aspect ratio 18–22), albeit from Avicel microcrystalline cellulose for their mixed-acid methodology.

As for sulfuric acid hydrolysis (similar acidic strength), Elazzouzi-Hafraoui et al. obtained widths of 12 to 27 nm by TEM analysis of cotton sCNCs.^53^ Our pCNCs appear to be thinner in comparison. The respective nanocrystal lengths are in good agreement, however, which is to be expected for CNCs from the same cellulose source. The discrepancy in particle width might be due to the significantly higher surface charge of our pCNCs compared to their sCNCs, which facilitates the dispersion and is bound to reduce the width of the single-crystal aggregates.

It can also be concluded that while the surface modification did not affect the hydrolysis reaction at all, it merely increases the acid consumption slightly due to neutralization of the surface phosphates.

WAXS analyses (Figure 5) showed that the cellulose materials retain their crystallinity during the modification process. Despite the presence of ammonia and increased pH during washing, no changes in the cellulose allomorph were observed as the diffraction patterns^54,55^ correspond to cellulose *I*_β_ throughout the entire process.

**Figure 6:**
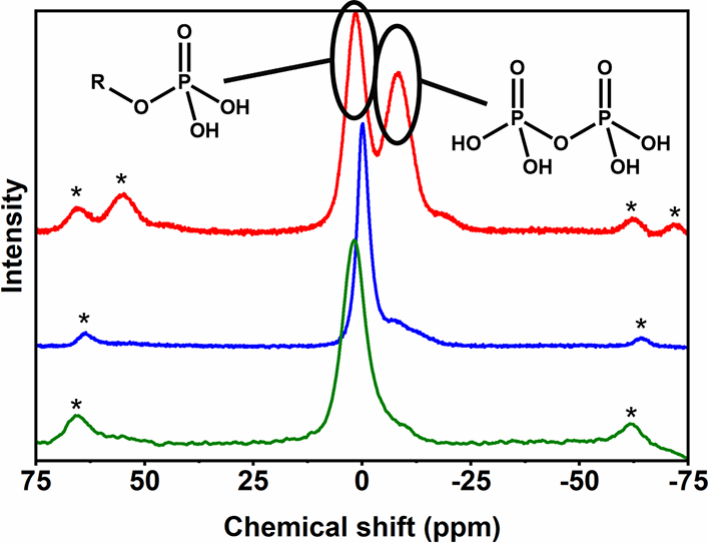
Solid-state ^31^P-MAS NMR spectra of the cellulose reaction mixture after modification reaction (red), hydrolyzed and washed phosphorylated pulp (blue), and dispersed pCNCs (green). Stars (*) denote spinning sidebands. Polyphosphates are formed during the modification but removed almost quantitatively in the following hydrolysis and washing steps.

In order to speciate the surface phosphate groups, solid-state ^31^P NMR was conducted (Figure 6). Evidently, the modification step leads to the formation of two separate phosphate species, which are most likely mono- and pyrophosphates. However, following the hydrolysis and washing, as well as the subsequent dispersion step, the signal for the pyrophosphate species is reduced significantly, leaving a large excess of phosphate half-ester groups.

The ^31^P NMR was measured under ^1^H-decoupling and is thus not quantitative. We found no linear relationship between peak area and P-content of the measured sample and a reference sample of ammonium phosphate. This could be due to the differences in density and proton concentrations which in effect change the spin saturation and relaxation behaviors. As such, while the ratios of peak areas from the same spectrum are comparable, quantitative comparisons between the separate spectra would be redundant. The peak area ratios for phosphate/pyrophosphate for the modified, hydrolyzed and washed, and dispersed samples are 50:50, 80:20, and 85:15, respectively, but given the width of the peaks resulting in considerable overlapping, these ratios need to be considered estimations. The crude ratio 80:20 does correspond to the findings of Zhao et al.,^56^ though.

It is certain that the phosphate is bound to the surface as the phosphorylated pulp (blue spectrum) was obtained after freeze-drying a suspension that showed no notable ionic strength (conductivity of 10 μS cm^–1^ at pH 4.5 for a 5.36 wt % dispersion). Highly soluble orthophosphate must therefore be absent. This result is in line with the previously reported modifications of both pCNCs^17^ and pCNF^24^ and highlights the successful modification and effective washing. A slight shift of the blue spectrum is due to the diligently washed pulp being fully protonated, whereas the modified fibers and the dispersed CNCs are neutralized.

Given the results from our NMR and FTIR (see, Figure S5) analyses, it is worth mentioning that no indication of the presence of phosphonate groups alongside the imparted phosphate groups has been found. Occasionally,^23,24,51^ the presence of phosphonate groups in this system has been speculated, which are claimed to be introduced to CNF alongside phosphate surface groups by the urea phosphate methodology. Although the covalent formation of phosphonates on anhydroglucose-containing substrates has indeed been established,^57−59^ this has been the result of a reaction with phosphorous acid, not phosphoric acid. Some articles ignore this difference in the substrate but assure the presence of phosphate species in the oxidation state (+III).^23,24,51^ We would like to state plainly that we consider it highly unlikely that phosphonates with the oxidation state (+III) could be formed from phosphoric acid (+V) in the absence of strong reducing agents—an assumption only strengthened by previously reported analyses^56^ and our NMR and FTIR data.

### Thermal Stability

**Figure 7:**
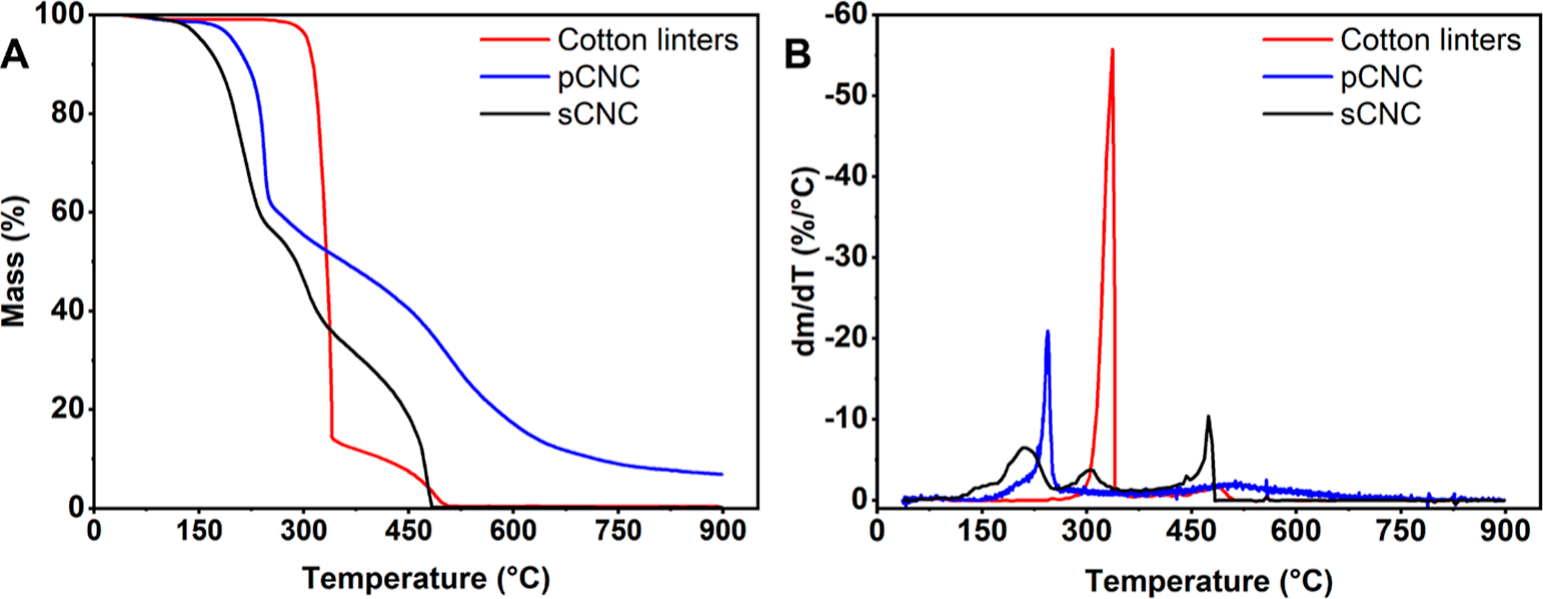
Thermal degradation behavior of cotton linters, produced pCNCs and sCNCs (A) and respective mass loss rates (B). The sample mass decreases to 95% at 160 °C (sCNCs), 207 °C (pCNCs), and 308 °C (cotton), with the strongest decline in mass occurring at 208, 244, and 337 °C, respectively.

The results of thermogravimetric analyses of phosphate- and sulfate-CNCs as well as unmodified cotton linters are shown in Figure 7. It clearly shows that CNCs carrying phosphate or sulfate half-esters have an earlier onset for degradation than native cellulose. However, the modification causes flame-retardant behavior that becomes evident in the significantly reduced mass loss rates at higher temperatures of the modified cellulosic materials. In the case of the pCNCs, the complete degradation occurs beyond 600 °C, more than 100 °C higher than the unmodified cotton or the sulfate CNCs. Furthermore, unlike with the pure carbohydrates or the sulfates, significant amounts of ash remain after the full combustion of the pCNCs. This is due to the formation of condensed polyphosphates, which are not volatile but remain in the crucible while sulfate degrades to form volatile SO_2_.

As such, the modified nanocrystals show reduced thermal stability given the earlier onset of mass loss, but both show flame-retardant behavior above 350 °C, that is, the mass loss is lower compared to the pristine cotton linters. In this context, the pCNCs show slightly increased thermal stability and significantly increased flame retardancy compared to sCNCs. These findings are in good accord with the already published data for sCNCs^60^ and pCNFs^25^ but do not match previous reports for pCNCs from phosphoric acid hydrolysis.^18,35,48,61^ However, similar data have been reported for previously isolated and consecutively pCNCs.^17,23^

The key influence on the degradation behavior seems to be the degree of substitution. As previously shown for sCNCs by Lin and Dufresne^60^ and for CNCs carrying both phosphate and sulfate half-esters by Vanderfleet et al.,^61^ higher degrees of substitution lead to earlier onsets in weight loss. This explains the comparable instability of highly charged pCNCs both in our analysis and in the literature.^17,23^ Furthermore, it follows from this comparison that the strength of the acidic surface groups has an influence on the degradation onset. A comparatively low degree of substitution with strongly acidic sulfate moieties promotes the degradation to a greater extent than the significantly higher degree of modification with phosphate groups.

## Conclusions

We have presented a reliable, straightforward process to produce high-charge pCNCs. Compared to the established phosphoric acid hydrolysis, we managed to significantly reduce the water consumption while achieving a much higher yield, reproducibility, and degree of substitution. Unlike the reported phosphorylation strategies for previously isolated CNCs, we managed to achieve comparable degrees of substitution while avoiding the energy-demanding initial isolation and drying of uncharged nanocrystals. Furthermore, we demonstrated that our pCNCs are equal in size and shape to CNCs isolated from cotton linters by gaseous HCl or sulfuric acid hydrolyses and equal in terms of surface charge and thermal degradation to pCNF obtained by urea/phosphate modification. As such, our pCNCs can be expected to show the same exceptional potential that has been demonstrated for pCNF in biomineralization, ion-exchange, and flame-retardancy applications. Nevertheless, further optimization of the stoichiometry of the modification reaction will be necessary before the potential for scaling up this synthesis can be harnessed.

## Supporting Information Available

Comparison to previously published phosphorylation protocols, cellulose discoloration during modification, conductometric titration curves, FTIR spectra, viscosity average DP values, and elemental composition of pCNCs and sCNCs (PDF)
