# Supplementary material for: Efficient Isolation Method for Highly Charged Phosphorylated Cellulose Nanocrystals
Source: Biomacromolecules. 2023 Feb 7;24(3):1318–28. doi: 10.1021/acs.biomac.2c01363 (PMC10015457; doi:10.1021/acs.biomac.2c01363)
Supplement: Supplementary file 1 — bm2c01363_si_001.pdf [file bm2c01363_si_001.pdf]

Supporting Information

Efficient Isolation Method for Highly Charged Phosphorylated Cellulose Nanocrystals

*Marcel Kröger[1], Olamide Badara[1], Timo Pääkkönen[1], Inge Schlapp-Hackl[1], Sami Hietala[2], Eero Kontturi[1]**

[1] Department of Bioproducts and Biosystems, Aalto University, FI-00076 Aalto, Finland

[2] Department of Chemistry, University of Helsinki, FI-00014, Finland

Table of Contents

Reported CNF phosphorylation protocols S2
Linear fit of the mass decrease during modification S3
Cellulose discoloration during modification/hydrolysis S4
Conductometric titration S5
FTIR S5
Elemental analysis S6
References S7

Reported CNF phosphorylation protocols

Table S1. Previously reported protocols for the phosphorylation of cellulose substrates for the isolation of pCNF compared to our modification protocol.

AGU	Phos-phate	Urea	Reagent	Pre-drying Temper-ature	Curing Temper-ature	Curing time	Charge or phosphate content [mmol/kg]	Refer ence
1	1.2	4.9	$(NH_4)_2HPO_4$	70 °C	150 °C	10 min	550[(a)]	
1	1.2	4.9	$(NH_4)_2HPO_4$	70 °C	150 °C	30 min	700[(a)]	
1	1.2	4.9	$(NH_4)_2HPO_4$	70 °C	150 °C	60 min	900[(a)]	
1	1.2	4.9	$(NH_4)_2HPO_4$	70 °C	150 °C	90 min	850[(a)]	
1	2	10	$(NH_4)_2HPO_4$	70 °C	150 °C	10 min	1450[(a)]	1
1	2	10	$(NH_4)_2HPO_4$	70 °C	150 °C	30 min	1820[(a)]	
1	2	10	$(NH_4)_2HPO_4$	70 °C	150 °C	60 min	1750[(a)]	
1	2	10	$(NH_4)_2HPO_4$	70 °C	150 °C	90 min	1300[(a)]	
1	0.29	0	NaH_2PO_4	105 °C	150 °C	60 min	90[(b)]	
1	0.56	0	NaH_2PO_4	105 °C	150 °C	60 min	173[(b)]	
1	0.9	0	NaH_2PO_4	105 °C	150 °C	60 min	611[(b)]	2
1	1.15	0	NaH_2PO_4	105 °C	150 °C	60 min	781[(b)]	
1	2.3	0	NaH_2PO_4	105 °C	150 °C	60 min	994[(b)]	
1	4.6	0	NaH_2PO_4	105 °C	150 °C	60 min	1136[(b)]	
1	0.63	3.24	$(NH_4)H_2PO_4$	-	165 °C	1.7 min	230[(b)]	
1	0.63	3.24	$(NH_4)H_2PO_4$	-	165 °C	2.5 min	790[(b)]	
1	0.63	3.24	$(NH_4)H_2PO_4$	-	165 °C	3.3 min	1230[(b)]	3
1	0.63	3.24	$(NH_4)H_2PO_4$	-	165 °C	5 min	1740[(b)]	
1	0.63	3.24	$(NH_4)H_2PO_4$	-	165 °C	10 min	2200[(b)]	
1	1.2	4.9	LiH_2PO_4	105 °C	150 °C	60 min	2440[(a)]	4

1	1.2	4.9	NaH_2PO_4	105 °C	150 °C	60 min	1480[(a)]	
1	1.2	4.9	(NH4)H2PO4	105 °C	150 °C	60 min	2210[(a)]	
1	1.2	4.9	NaH_2PO_4	105 °C	150 °C	60 min	410[(a)]	
1	1.2	4.9	$(NH_4)_2HPO_4$	105 °C	150 °C	60 min	2930[(a)]	
1	0.5	2	$(NH_4)_2HPO_4$	70 °C	150 °C	15 min	2080[(a)]	
1	0.5	2	$(NH_4)_2HPO_4$	70 °C	150 °C	30 min	2560[(a)]	5
1	0.5	2	$(NH_4)_2HPO_4$	70 °C	150 °C	60 min	1920[(a)]	
1	1	4.7	H_3PO_4 : NaH_2PO_4 1:1	-	105C	72h	1000[(b)] 1920[(a)]	This work)

(a) charge content determined by titration

(b) phosphate content determined via molybdenum blue spectrophotometry[3]

(c) phosphate content determined via elemental analysis[2] (this work)

Linear fit of the mass decrease during modification

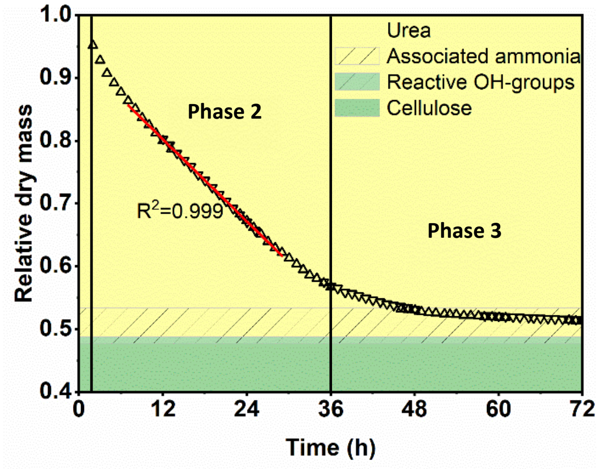

Figure S1. Linear regression for the mass decrease associated to the decomposition of urea (Phase 2). The fitted slope is -0.0109 h^{-1} which is equivalent to a rate constant k = 3.61 mol/s (see, equation S1).

The kinetics of a 0-order reaction are represented in equation S1:

$$[A] = [A]_0 - kt \tag{S1a}$$

$$k = \frac{[A]_0 - [A]}{t} \tag{S1b}$$

$[A]$ and $[A]_0$ being the current and initial concentration of the decaying compound A, t being the time and k being the rate constant.

Cellulose discoloration during modification/hydrolysis

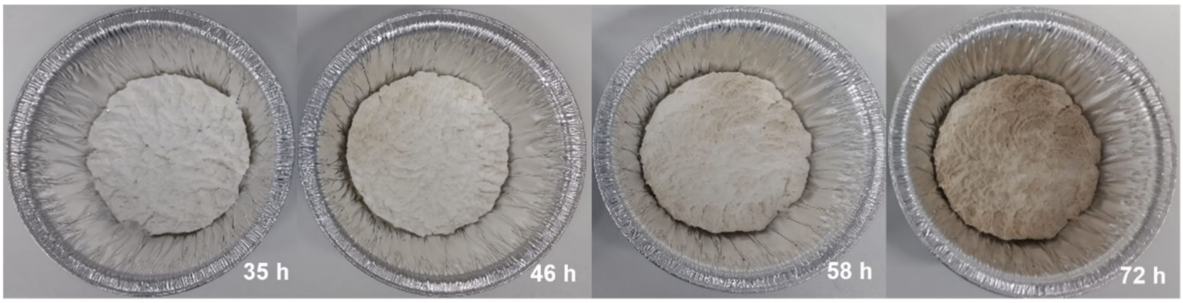

Figure S2. Cellulose discoloration during the modification reaction. Pictures taken after 35 h, 46 h, 58 h and 72 h.

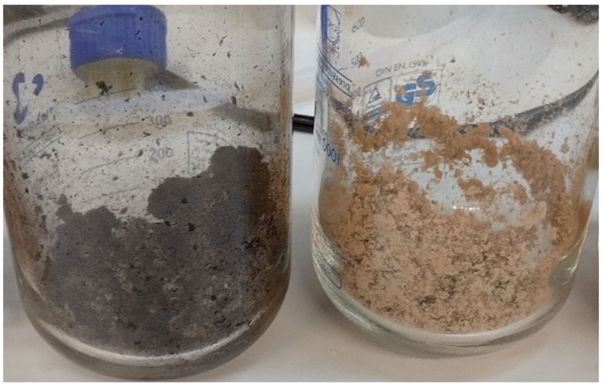

Figure S3. Blackening of modified cellulosic samples upon contact with HCl gas after 12h of modification reaction (left) compared to no observed effect for a sample after completion of the modification reaction after 72 hours (right)

Viscosity-average degree of polymerization

According to ISO standard 5351:2004, the limiting viscosity of samples of the unmodified cotton linters and samples after modification, hydrolysis and washing was determined after dissolution in cupriethylene diamine solution (CED, CAS 14552-35-3, SCAN 16:62, FF-Chemicals). An estimation of the degree of polymerization based on the obtained limiting viscosities was done by means of the Mark Houwink equation as established by Marx Figini[6] and supported by Ucar and Balaban[7]. The viscosity measurements were done after 30-45 min after adding the CED solution to the cellulosic samples to avoid degradation. Within this timeframe, unmodified cotton linters and modified, hydrolyzed and washed pulp dissolved completely, whereas modified cotton linters pre-hydrolysis did not dissolve even after extensive washing.

The relations used to calculate limiting viscosities and viscosity-average degrees of polymerizations are shown as equation S2a below:

$$\begin{aligned}[\eta] = \frac{1}{c}(&0.035451228(\log([\eta]_{rel} - 1))^6 \\ &- 0.069902813(\log([\eta]_{rel} - 1))^5 \\ &- 0.12206756\,(\log([\eta]_{rel} - 1))^4 \\ &+ 0.29198129\,(\log([\eta]_{rel} - 1))^3 \\ &+ 1.1082113\,(\log([\eta]_{rel} - 1))^2 \\ &+ 1.470473\,(\log([\eta]_{rel} - 1)) + 0.7895077)\end{aligned} \qquad \text{(S1a)}$$

$$[\eta] = K\,\mathrm{DP}_v^{\alpha}$$

$$K = 2.28\ and\ \alpha = 0.76\ for\ DP > 950 \qquad \text{(S2b)}$$

$$K = 0.42\ and\ \alpha = 1\ for\ DP < 950$$

$[\eta]$ being the limiting viscosity of the dissolved sample, c the cellulose concentration in the CED solution, $[\eta]_{rel}$ the relative viscosity of the CED solution as determined experimentally, and K and α the Staudinger-Mark-Houwink constants for cotton cellulose[6] and DP_v the viscosity-average degree of polymerization.

Table S2. Average values for relative and limiting viscosities and calculated viscosity-average degrees of polymerization.

**Sample**	***Mass (g)***	***Time (s)***	$[\eta]_{rel}$	$[\eta]$	$\mathbf{DP}_v$
cotton linters 1	0.1549	57.66 ± 0.17	6.060 ± 0.018	752.0 ± 1.2	1790 ± 3
cotton linters 2	0.1516	55.84 ± 0.44	5.869 ± 0.046	754.6 ± 3.3	1797 ± 8
phosphorylated pulp 1	0.4070	20.64 ± 0.21	2.170 ± 0.022	109.9 ± 1.6	163.9 ± 3.2
phosphorylated pulp 2	0.6154	26.78 ± 0.19	2.815 ± 0.020	101.4 ± 0.8	147.4 ± 1.5

As can be seen in Table S2, the treatment of the fibers results in a significant decrease of the degree of polymerization to less than a tenth of the native fibers.

Conductometric titration

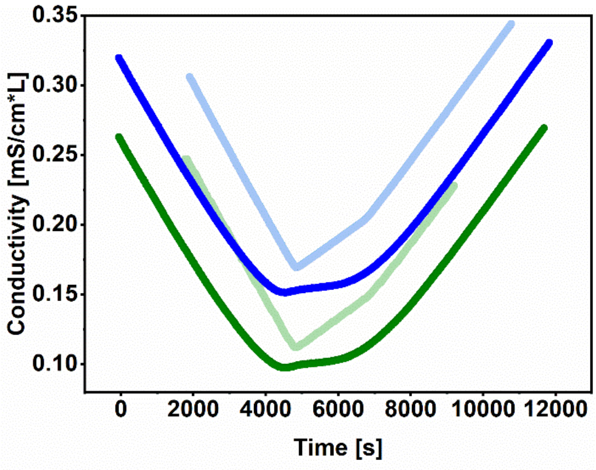

Figure S4. Conductometric titration curves of the produced pCNCs recorded in 0.5mM (green) and 1.5mM NaCl solutions (blue) compared to titration curves of the same amount of sodium phosphate in 0.5 mM (pale green) and 1.5 mM NaCl solutions (pale blue). The samples contain 0.5 mmol HCl (neutralized after 3000s) and 300 mg protonated pCNCs (0.3 mmol phosphate according to elemental analysis, neutralized after 4800s and 6600s) and were titrated with 0.1M NaCl at 0.1mL/min. Conventional analysis by linear regressions of the three segments yields a "charge content" of 1920mmol/g.

FTIR

ATR-FTIR spectra were recorded on a Perkin Elmer Spectrum Two FT-IR in the range of 400 cm^{-1} -4000 cm^{-1}. The displayed spectra were baseline-corrected using the Perkin Elmer Spectrum 10.6.2 software.

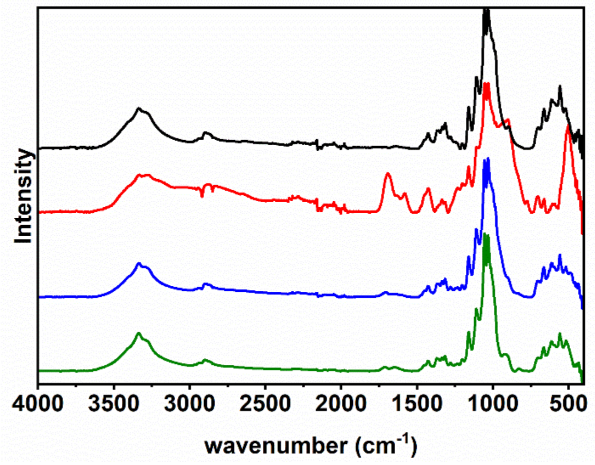

Figure S5. FTIR spectra of Whatman1 filter paper (black), modified cellulose (red), hydrolyzed and washed phosphorylated pulp (blue) and dispersed CNC (green). The band commonly

attributed to phosphate groups at 900 cm^{-1} is present in the unmodified, phosphate free filter paper as well.

Elemental analysis

Table S3. Elemental composition of filter paper and produced pCNC

Element	Whatman 1	pCNC	sCNC
**C**	44.19 %	37.48 %	40.45 %
**H**	6.31 %	5.62 %	5.88 %
**N**	0	0.42 %	0
**P**	-	3.1 %	
**S**			0.62 %

References

(1) Ghanadpour, M.; Carosio, F.; Larsson, P. T.; Wågberg, L. Phosphorylated Cellulose Nanofibrils: A Renewable Nanomaterial for the Preparation of Intrinsically Flame-Retardant Materials. *Biomacromolecules* **2015**, *16* (10), 3399–3410. https://doi.org/10.1021/acs.biomac.5b01117.

(2) Naderi, A.; Lindström, T.; Flodberg, G.; Sundström, J.; Junel, K.; Runebjörk, A.; Weise, C. F.; Erlandsson, J. Phosphorylated Nanofibrillated Cellulose: Production and Properties. *Nord. Pulp Pap. Res. J.* **2016**, *31* (1), 20–29. https://doi.org/10.3183/npprj-2016-31-01-p020-029.

(3) Noguchi, Y.; Homma, I.; Matsubara, Y. Complete Nanofibrillation of Cellulose Prepared

by Phosphorylation. *Cellulose* **2017**, *24* (3), 1295–1305. https://doi.org/10.1007/s10570-017-1191-3.

(4) Rol, F.; Sillard, C.; Bardet, M.; Yarava, J. R.; Emsley, L.; Gablin, C.; Léonard, D.; Belgacem, N.; Bras, J. Cellulose Phosphorylation Comparison and Analysis of Phosphorate Position on Cellulose Fibers. *Carbohydr. Polym.* **2020**, *229* (August 2019), 115294. https://doi.org/10.1016/j.carbpol.2019.115294.

(5) Messa, L. L.; Faez, R.; Hsieh, Y.-L. Phosphorylated Cellulose Nanofibrils from Sugarcane Bagasse with PH Tunable Gelation. *Carbohydr. Polym. Technol. Appl.* **2021**, *2* (March), 100085. https://doi.org/10.1016/j.carpta.2021.100085.

(6) Marx-Figini, M. Significance of the Intrinsic Viscosity Ratio of Unsubstituted and Nitrated Cellulose in Different Solvents. *Die Angew. Makromol. Chemie* **1978**, *72* (1), 161–171. https://doi.org/10.1002/apmc.1978.050720114.

(7) Uçar, G.; Balaban, M. Accurate Determination of the Limiting Viscosity Number of Pulps. *Wood Sci. Technol.* **2004**, *38* (2), 139–148. https://doi.org/10.1007/s00226-003-0218-0.
